# Rapid regulation of excitation energy in two pennate diatoms from contrasting light climates

**DOI:** 10.1007/s11120-018-0558-0

**Published:** 2018-07-14

**Authors:** Allen K. Derks, Doug Bruce

**Affiliations:** Department of Biological Sciences, Brock University, 1812 Sir Isaac Brock Way, Saint Catharines, ON L2S 3A1 Canada

**Keywords:** Excitation pressure, Light harvesting, Non-photochemical quenching, Photoprotection

## Abstract

Non-photochemical quenching (NPQ) is a fast acting photoprotective response to high light stress triggered by over excitation of photosystem II. The mechanism for NPQ in the globally important diatom algae has been principally attributed to a xanthophyll cycle, analogous to the well-described qE quenching of higher plants. This study compared the short-term NPQ responses in two pennate, benthic diatom species cultured under identical conditions but which originate from unique light climates. Variable chlorophyll fluorescence was used to monitor photochemical and non-photochemical excitation energy dissipation during high light transitions; whereas whole cell steady state 77 K absorption and emission were used to measure high light elicited changes in the excited state landscapes of the thylakoid. The marine shoreline species *Nitzschia curvilineata* was found to have an antenna system capable of entering a deeply quenched, yet reversible state in response to high light, with NPQ being highly sensitive to dithiothreitol (a known inhibitor of the xanthophyll cycle). Conversely, the salt flat species *Navicula* sp. 110-1 exhibited a less robust NPQ that remained largely locked-in after the light stress was removed; however, a lower amplitude, but now highly reversible NPQ persisted in cells treated with dithiothreitol. Furthermore, dithiothreitol inhibition of NPQ had no functional effect on the ability of *Navicula* cells to balance PSII excitation/de-excitation. These different approaches for non-photochemical excitation energy dissipation are discussed in the context of native light climate.

**Electronic supplementary material:**

The online version of this article (10.1007/s11120-018-0558-0) contains supplementary material, which is available to authorized users.

## Introduction

Photosynthetic organisms have a compromising relationship with their light environment: there is the obligatory need for photons, but a rapid increase in light intensity can disrupt photostasis with potentially deleterious consequences. Heightened excitation pressure on photosystem (PS) II develops when the amount of light energy absorbed by the antenna system and transferred to the PSII reaction centre exceeds what can be dissipated via the relatively slower processes of photochemistry and forward electron transport (Huner et al. 1998). Over excitation of PSII can lead to the formation of reactive oxygen species, photodamage, and ultimately inactivation of PSII (photoinhibition). Over acidification of the lumen and over reduction in the redox status of the photosynthetic electron carriers are physiological indicators of high PSII excitation pressure (Derks et al. 2015).

Diatoms account for a significant proportion of all aquatic primary production (Smetacek 1999); these ubiquitous algae can also be important constituents of terrestrial communities (Kirkwood and Henley 2006; Schulz et al. 2016). The growth rate and photosynthetic capacity of diatoms are less affected by light fluctuations than other groups of algae when measured in the laboratory (Mitrovic et al. 2003) or in the field (Petersen et al. 1998). Diatoms also show a higher resistance to PSII photoinactivation when compared to other algal groups (Key et al. 2010).

Non-photochemical quenching (NPQ) is a process whereby PSII excess excitation energy is dissipated non-radiatively as heat. NPQ is readily observed in vivo as a drop in maximal PSII fluorescence during high light (HL) illumination. Quenching can originate from within the antenna system or within the PSII reaction centre (Derks et al. 2015). qE describes a fast acting (induced within a few seconds of HL illumination) form of NPQ which is dependent on ∆pH (the trans-thylakoid proton gradient) energization of the thylakoid. qE in higher plants has been intensely investigated, though there remains active discussion about many of the mechanistic details (reviewed by Derks et al. 2015; Ruban 2016). There is solid evidence that a major component of the antenna-based qE mechanism in higher plants involves a ∆pH triggered, xanthophyll cycle-modulated change in light harvesting complex (LHC) II macro-organisation that creates Chl*a*–Chl*a* and/or Chl*a*–carotenoid associations with short-lived excited states (Horton et al. 2005; Miloslavina et al. 2008; Andreeva et al. 2009; Ruban et al. 2012). Xanthophyll cycle dependent qE is hereafter referred to as qE_XC_.

Diatom NPQ has been principally attributed to qE_XC_ mediated by the diadinoxanthin (DD)/diatoxanthin (DT) cycle, whereby acidification of the lumen activates DD de-epoxidase conversion of DD to DT. qE_XC_ induction in diatoms seems to require both ΔpH and active DD de-epoxidation (Lavaud 2007; Goss and Jakob 2010). Dithiothreitol (DTT), a well-known inhibitor of plant violaxanthin de-epoxidase (Hieber et al. 2002), also inhibits DD de-epoxidation in diatoms (Grouneva et al. 2008). Ammonia, acting as a membrane permeable proton scavenger, has been shown to inhibit diatom qE and dissipate pre-existing qE (Ruban et al. 2004). Upon termination of HL conditions, NPQ in diatoms generally relaxes more slowly than in higher plants, which has been attributed to a strong dependence on DT epoxidation (Ruban et al. 2004; Lavaud 2007). LHCs of diatoms are composed of fucoxanthin chlorophyll proteins (FCPs), heavily enriched in the xanthophyll fucoxanthin (Fx) and containing Chl*c*s as a substitute for Chl*b*. FCPs are structurally and functionally heterogeneous, differing in protein and pigment composition, and oligomeric organization within the thylakoid, with some FCP complexes being more tightly coupled to PSII or PSI while other FCP complexes form aggregates which more weakly associate with the photosystems (Lavaud et al. 2003; Lepetit et al. 2010; Grouneva et al. 2011; Gundermann et al. 2013; Gardian et al. 2014; Gundermann and Büchel 2014). The stress-related Lhcx proteins serve to modulate the NPQ response (Zhu and Green 2010; Bailleul et al. 2010; Lepetit et al. 2017). Investigators have variably implicated FCP supramolecular reorganisation and Chl*a*-carotenoid and Chl*a*–Chl*a* interactions in diatom qE (Frank et al. 1996; Ruban et al. 2004; Miloslavina et al. 2009; Chukhutsina et al. 2014; Wahadoszamen et al. 2014; Goss and Lepetit 2015; Giovagnetti and Ruban 2017). Working NPQ models have advocated that there are two distinct types of quenching sites in diatoms (Miloslavina et al. 2009; Chukhutsina et al. 2014; Lavaud and Goss 2014; Derks et al. 2015; Goss and Lepetit 2015). pH-induced decoupling in inter-PSII monomer energy transfer has been suggested to facilitate non-photochemical quenching (Yokono et al. 2015). PSII cyclic electron transfer can be another important photoprotective mechanism in some diatoms, enabling PSII to dynamically react to very rapid changes in irradiance independent of qE_XC_ (Lavaud et al. 2002, 2007).

The NPQ responsiveness of a diatom species/strain can be regarded as a functional marker for its light niche, and may explain the spatial and geographic distribution of diatoms in the modern oceans (Lavaud et al. 2007, 2016; Goss and Jakob 2010; Giovagnetti et al. 2014; Juneau et al. 2015). Lavaud et al. (2007) initially compared the photoprotective behavior of planktonic diatom species isolated from marine habitats with different water mixing dynamics after being cultured under identical light regimes. Species from stable light environs (open ocean and coastal waters) had the lowest NPQ capacity, whereas the species from a highly dynamic underwater light climate (estuary) exhibited the highest NPQ capacity (Lavaud et al. 2007). Coastal diatoms exposed to gradual changes in irradiance representative of diel light cycles exhibit a slow inducing, moderate amplitude NPQ; yet perform a fast inducing, higher magnitude NPQ response when exposed to mixing-related changes in irradiance (Giovagnetti et al. 2014). The photophysiological responses of benthic diatoms can vary according to their specific habitats and morphology, including cell motility (Barnett et al. 2015; Juneau et al. 2015; Blommaert et al. 2017, 2018). The PSII repair cycle has been shown to work in close coordination with the NPQ responses of species originating from both a fluctuating and a more stable light climate (Lavaud et al. 2016), confirming the fundamental role of NPQ in diatom photophysiology.

The objective of this study was to compare the fast-acting photoprotective responses of two pennate diatom species which originate from different benthic light climates. *Nitzschia curvilineata* was collected from the Long Island Sound shoreline in Connecticut, USA (Provasoli-Guillard National Center for Marine Algae and Microbia. Bigelow Laboratory for Ocean Sciences 2017). This species was hypothesized to exhibit a robust NPQ response over a broad range of HL intensities, with NPQ induction/relaxation kinetics in the time frame of wave-induced mixing (seconds to minutes). *Navicula* sp. 110-1 was isolated from the soil surface of the Great Salt Plains salt flat in Oklahoma, USA (Kirkwood and Henley 2006). The salt flat can be considered a more photostable environment than the shoreline; fluctuations in light would be primarily due to atmospheric and diel effects, not by mixing through a water column. *Navicula* was hence hypothesized to exhibit a less dynamic NPQ response as compared to its shoreline counterpart. The NPQ processes in these two species were characterized in whole cells by using: (i) 77 K steady state absorption and emission spectroscopy to measure HL elicited changes in the absorbance and energy transfer landscapes of the thylakoid, (ii) variable fluorescence to monitor in real-time PSII excitation energy conversion during HL transitions, and (iii) DTT and NH_4_Cl treatments to estimate the contribution of qE_XC_ towards non-photochemical energy dissipation.

## Methods

### Culturing and sample preparation

*Nitzschia curvilineata* (CCMP 555) and *Navicula* sp. 110-1 (CCMP 2566) were purchased from NCMA (Provasoli-Guillard National Center for Marine Algae and Microbiota, Bigelow Laboratory for Ocean Sciences, East Boothbay, Maine, USA). Cultures were grown in an oligotrophic artificial sea water medium based upon the ESAW (Berges et al. 2001) and F/2 (Guillard 1975) formulations with silicate added in the form of Na_2_SiO_3_ × 9H_2_O to a final concentration of 200 μM. Cultures were grown in 1 L polycarbonate square bottles vented through the top caps with 0.2 μm polytetrafluoroethylene filter membranes. Culture bottles were placed in an 18 °C water bath and illuminated from the sides by GE Daylight 6500 K compact fluorescent lamps. Photon flux reaching inside the culture bottles was 50 ± 10 μmol m^−2^s^−1^, measured with a LI-189 lightmeter (Li-Cor, USA). Cultures were grown under an 18 h day/6 h night cycle and gently shaken twice diurnally. Cultures were maintained at a Chla concentration of approximately (~) 0.05 mg/mL by regularly diluting the cultures with fresh growth media. Cells were cultured under these continuous growth conditions for at least 2 months after receipt from NCMA before being used for physiological measurements. Cell samplings were collected from the bottom of the culture bottles at times corresponding to midday of the photoperiod and were treated to the following routine immediately prior to experimentation: (1) Cell aliquots were dark adapted at 18 °C for 15 min, (2) cells were concentrated by spinning at low RPM for 30 s in the dark. Supernatant was discarded, (3) cells were gently suspended in fresh growth media supplemented with 10 mM KHCO_3_ to an equivalent Chl*a* concentration of 0.1 mg/mL for 5 min at 18 °C in the dark. Bicarbonate was added to prevent CO_2_ limitation in the concentrated cell samples. The growth media + 10 mM KHCO_3_ solution was augmented with DTT or NH_4_Cl as required. All solutions were made fresh daily, (4) cells were concentrated by spinning at low RPM for 30 s in the dark. Supernatant was removed to achieve desired Chl*a* concentration.

### Pigment analysis

Pigment extractions were performed under low light and on ice. Cell sample concentrations (mg/mL Chl*a*) were calculated from methanol extracts using the extinction coefficient of Porra et al. (1989). Determination of cell pigment composition was adapted from Seely et al. (1972) using the extinction coefficients and solvent ratios described therein: cells were collected via centrifugation and then rinsed in distilled water, the washed cell pellet was suspended in dimethyl sulfoxide for 15 min, the extract was collected, and then the pellet was suspended in acetone for 5 min, the extract was collected, and then the pellet was rehydrated with distilled water, the acetone treatment was repeated, the pellet was suspended in 1:1 acetone: methanol (volume: volume) for 5 min, the extract was collected. An absorption spectra of the pooled extract was collected using a DW-2 scanning spectrophotometer (Aminco, USA) in split mode, 1.0 nm slit width controlled by an OLIS acquisition system (OLIS, Bogart, GA, USA). Optical density values were kept ≤ 0.3.

### Low temperature spectroscopy

#### Absorption spectra

The DW-2 spectrophotometer described above was reconfigured *in lab* to be used in a surface reflectance mode. The measuring beam was optically coupled into one leg of a bifurcated fibre optic bundle; the common end of the bundle was used to deliver the measuring light to the sample and collect the reflected signal light; the other leg of the bundle was used to deliver the signal light to the photomultiplier detector. Another bifurcated fiber optic bundle was used in the same manner for the reference signal. The positioning of the fibre bundles was optimized to negate any positional effects from the individual fibres on light collection and emission from the bundles. Three-eighths inch diameter circles of P8 filter paper (Fischer Scientific) were affixed to one end of 1/2 inch diameter steel rod cold fingers using a small amount of thermal grease. Samples were exposed to the appropriate light regime, loaded onto the filter paper, then flash frozen in liquid N_2_ under the corresponding light conditions. Filter paper plus the same suspension media used with the sample was used as the reference blank. A custom sample holder held the cold fingers above a liquid N_2_ reservoir and coupled the fibre optic bundles. Actual temperature of the sample in this setup was 77–80 K as measured with a cryo-silicon diode (model 9600-1; Scientific Instruments Inc, West Palm Beach, Fla., USA). The samples were thermally stable for the duration of spectra collection. Optical density values were kept ≤ 0.3.

#### Emission spectra

Excitation light was provided by a 470 nm LED (Cree SiC Technology, Durham, N.C., USA) filtered through a 480 nm 12.5 nm FWHM bandpass filter. Samples of 0.18 mg/mL Chl*a* were loaded into 3/16 inch diameter, 1 mm deep wells of PVC sample sticks, exposed to the desired light regime, then flash frozen in liquid N_2_ under the same light condition. The sample sticks were positioned within the narrow stem of a custom Pyrex dewar and submerged in liquid N_2_. A stream of pressurized air was used to prevent condensation buildup. Sample emission was collected 90° to excitation by a fibre bundle protected with a 660 nm Schott long-pass filter. The fibres of the distal-to-sample end of the bundle were arranged so as to form the entrance slit to the detection side monochromator (Triax 320; Jobin Yvon Inc, Edison, NJ, USA). The detector was a liquid N_2_ cooled CCD array (Symphony; Jobin Yvon). The system was calibrated to the spectral lines of a neon lamp and had a detection bandwidth resolution of 1.5 nm FWHM. Background emission spectra collected under the same experimental conditions were subtracted from the raw spectra, and then the resulting spectra were corrected using an instrument response function created from the experimentally collected emission spectra of the fluorescent dye LDS-751 and the published corrected spectra of said dye (Lakowicz 2006).

### PAM (pulse amplitude modulated) fluorometry

#### Definition of fluorescence parameters

*Fmdark* is the maximal fluorescence level attained in dark adapted cells during the application of a saturating light pulse. *Fm′* is the maximal fluorescence level attained in cells during the application of a saturating light pulse. *Fodark* is the basal fluorescence level attained in dark adapted cells prior to application of a saturating light pulse (measured with the application of weak far-red light). *Fo′* is the basal fluorescence level attained in cells after the application of a saturating light pulse during illumination (measured without actinic illumination in the presence of weak far-red light). *Ft* is the transient fluorescence level prior to application of a saturating light pulse.

The maximum quantum efficiency of PSII photochemistry:

$$\Phi {\text{PSII}}~=\frac{{~Fmdark - Fodark}}{{Fmdark}}$$

The photochemical quenching parameter:

$$qP~=~\frac{{Fm^{\prime} - Ft}}{{Fm^{\prime} - Fo^{\prime}}}~$$

The extent of Stern–Volmer non-photochemical fluorescence quenching (Bilger and Björkman 1990):

$${\text{NPQ}}~=\frac{{Fmdark - Fm^{\prime}}}{{Fm^{\prime}}}$$

The quantum yields of PSII energy flux (Hendrickson et al. 2004):

$$\phi {\text{PSII}}~=\frac{{Fm^{\prime} - Ft}}{{Fm^{\prime}}}$$

$$\phi {\text{NPQ}}=~\frac{{Ft}}{{Fm^{\prime}}} - \frac{{Ft}}{{Fmdark}}$$

$$\phi {\text{NO}}~=\frac{{Ft}}{{Fmdark}}$$

#### Experimental setup

Dark adapted samples of 3 mg/mL Chl*a* were placed into 11 mm diameter × 2 mm high silicon walled sample chambers and covered with a #1 20 × 20 mm coverslip (Fischer Scientific). The sample chamber was placed into a custom built temperature controlled sample holder held at 18 °C. A multifurcated fibre optic bundle was used to deliver the various light sources to the sample and collect the fluorescence signal from the sample. The common terminus of the fibre optic bundle was coupled to the sample from above. The fibres in the common terminus of the bundle were mixed to minimalize small scale positioning effects within the sample. The pulsed measuring light was provided by a 470 nm LED (Cree SiC Technology). Fluorescence emission was selected for amplification with a 680 nm long-pass Schott glass filter. A PAM 101 chlorophyll fluorometer (Heinz Walz GmbH, Effeltrich, Germany) powered by an external battery was used to modulate the measuring light and lock-in amplify the fluorescence signal. For quasi-dark measurements the measuring light was pulsed at 1.6 KHz giving an integrated photon flux reaching the sample of no more than 0.50 µmol m^−2^ s^−1^. For measurements under illumination conditions the measuring light was pulsed at 100 KHz to increase kinetic resolution. Multiturnover saturating light pulses (5000 µmol m^−2^ s^−1^, 300 ms) were used to momentarily close PSII reaction centres for the collection of *Fmdark* and *Fm′*. Multiturnover saturating light pulses and actinic light were provided by a solid state triggered high power dual channel LED (neutral white; LED Engine Inc) allowing independent control over intensity and duration for the saturating light pulses and actinic light. These white light sources were passed through a 675 nm high performance short-pass filter (Edmund Optics, Barrington, N.J., USA). Photon flux from the 100 KHz measuring light was included when measuring actinic light photon flux. Far-red light for collection of *Fodark* and *Fo′* was provided by a solid state triggered 720 nm LED (LED Engine Inc). The saturating light pulse/actinic light and far-red light modules were designed and built *in lab* (A.D.). The PAM 101 electronics were modified to allow remote triggering of measuring light and pulse frequency selection. Output signal voltage from the PAM 101 was digitized with a 12 bit 250,000 samples/second analogue/digital converter (PCI-DAS1000; Measurement Computing, Norton, MA., USA). Software developed *in lab* (A.D.) was used to control triggering of all the light sources and to collect and process the fluorescence signal in real-time.

### Effective PSII absorbance cross section measurements

Effective PSII absorbance cross section (σPSII) measurements were performed following the approach of Falkowski and Chen (2003). Cells were dark adapted for 15 min, then resuspended in growth media + 10 mM KHCO_3_ doped with 4 µM final concentration DCMU (3-(3,4-dichlorophenyl)-1,1-dimethylurea) for 5 min. Chl*a* concentration, sample holder and light delivery to the sample as described in 2.4.2. Measuring pulses (500 µs duration, 500 µs apart, controlled by a fast response solid state relay) were provided by a 470 nm LED (Cree SiC Technology) screened with a 480 nm 12.5 nm FWHM bandpass filter in combination with a B440 Schott glass filter. The fluorescence detection photodiode (Si PIN type; Thor Labs, Inc, Newton, NJ, USA) was protected with a 680 nm long-pass Schott glass filter in combination with a 680 nm 12.5 nm FWHM bandpass filter. Photodiode current was amplified and converted to voltage using op-amp circuitry and then digitized using the PCI-DAS1000. Software developed *in lab* (A.D.) was used for pulse triggering and data acquisition.

### Data transformation

Graph generation, statistical analysis, and data fitting were performed using ORIGIN software (OriginLab Corp, Northampton, Mass., USA).

## Results and discussion

### Characterization of the excited state landscapes of the thylakoid

#### Basal light harvesting

Both diatom species were considered acclimated to the same light climate since they were cultured under identical conditions for a multitude of generations. Nevertheless, the salt flat species *Navicula* produced a smaller FCP antenna size for PSII than the shoreline species *Nitzschia. Navicula* cells had fewer accessory pigments per Chl*a* and had a smaller effective PSII absorbance cross section (σPSII) when measured with FCP excitation (Table 1).

**Table 1:** Light harvesting summary for dark adapted cells

Species	(Chl*c*)^a^/(Chl*a*)	(Car)^b^/(Chl*a*)	σPSII (*A*^2^/quanta)	Fmdark/(Chl*a*) (arb. units)
*Nitzschia*	0.503 ± 0.054	1.64 ± 0.09	859.6 ± 73.5	3400 ± 304
*Navicula*	0.411 ± 0.020	1.03 ± 0.05	550.9 ± 19.5	2107 ± 368

Pigment concentrations were determined photometrically from solvent extracts. 470 nm probing excitation was used for measuring σPSII and Fmdark. Means ± 1 SD from three separate cultures

^a^Chl*c* includes both Chl *c*_1_ and *c*_2_

^b^All carotenoids including Fx, DD, DT, and β-carotene

**Fig. 1:**
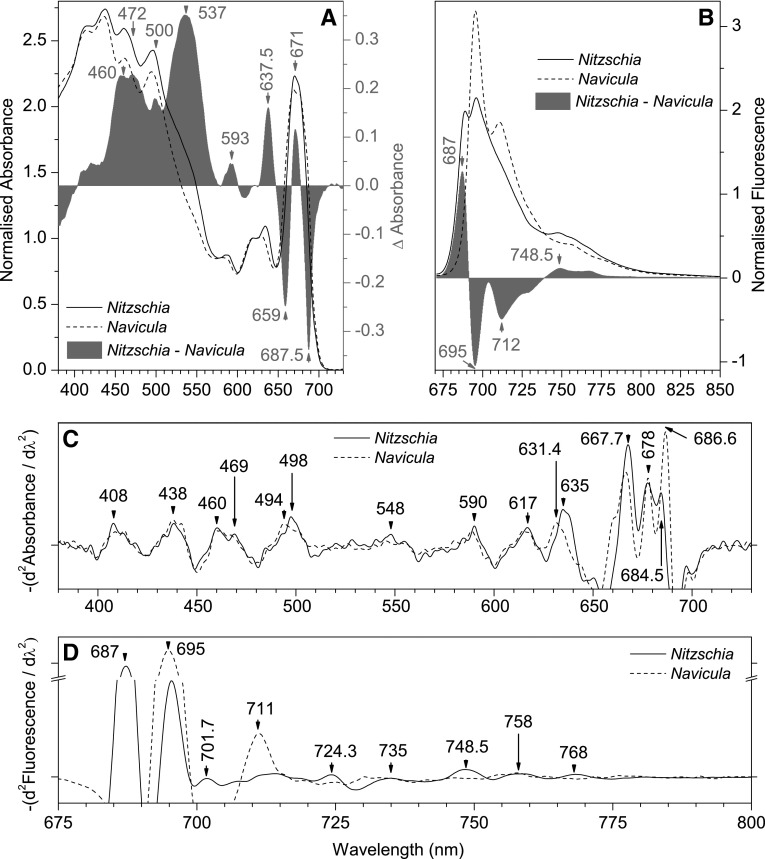
77 K absorption and emission spectra from dark adapted *Nitzschia* (solid line) and *Navicula* (dashed line) cells. **a** Absorption spectra normalized to Chl*a* 623 nm peak. Spectra are the average of three samplings from four separate cultures (*n* = 12). The difference between the normalized spectra (*Nitzschia* minus *Navicula*) is shown in grey; difference peaks are labeled in grey. **b** 77 K Emission spectra (470 nm excitation) normalized to integrated emission (from 670 to 850 nm). Spectra are the mean from three separate cultures. The difference between the normalized spectra (*Nitzschia* minus *Navicula*) is shown in grey; difference peaks are labeled in grey. **c** Inverted second derivatives (5.0 nm integration interval) of the absorption spectra in **a**, with select positions labeled. **d** Inverted second derivative (5.0 nm integration interval) of the emission spectra in **b**, with select positions labeled

Low temperature absorption spectra of dark adapted cells (Fig. 1a) were used to investigate inter-specie differences in in situ pigment composition/organization. To limit distortions in the absorption spectra that could be caused by pigment packaging effects (Stuart et al. 1998, 2000) or Chl*a* aggregation (Sauer et al. 1966; Ruban et al. 1997), the spectra were normalized to the 623 nm Qx Chl*a* peak. Enhanced 77 K cellular absorbance in *Nitzschia* (relative to bulk Chl*a*) was principally assigned to Chl*c* (at ~ 460, 593, 638 nm) and carotenoids (at ~ 472, 500, 537 nm) using 77 K absorption spectra of isolated pigments (Online Resource 1), absorbance maxima assigned by the FCP model of Premvardhan et al. (2010), and the remarks of Lavaud (2007). The broad peak of enhanced absorbance in *Nitzschia* centred at ~ 537 nm was assigned to a population of Fx molecules with differing degrees of bathochromic shifting.

Whole cell low temperature emission spectra collected with FCP excitation were used to investigate energy transfer in dark adapted cells. *Nitzschia* emitted maximally at ~ 689 and ~ 696 nm (derivative resolved bands at 687 and 695.5 nm), whereas *Navicula* cells emitted maximally at ~ 695 nm with a secondary peak at 711 nm (Fig. 1b, d) but lacked the ~ 685 nm CP43 peak typical of all PSII (Andrizhiyevskaya et al. 2005). Based on overlap in the 77 K emission spectra of isolated *Phaeodactylum tricornutum* FCP complexes (Lepetit et al. 2007) and purified *Chaetoceros gracilis* PSII (Nagao et al. 2010), the 687 nm emission band observed in *Nitzschia* cells would have contributions from both FCP and PSII. The ratio of F689:F696 varied between *Nitzschia* cultures (data not shown). Dense, intermittent light:dark, or red light grown cultures of *P. tricornutum* exhibit a strong emission peak at ~ 710 nm (Lavaud and Lepetit 2013; Herbstová et al. 2015, 2017), with F710:F687 ratio positively correlating with NPQ capacity (Lavaud and Lepetit 2013). The F710 emitter serves as an antenna for PSII and was located within a red-shifted FCP composed of oligomeric Lhcf15 (Herbstová et al. 2015, 2017). Even though diatom PSI cores have a species-dependent 77 K emission peak from around 710 to 720 nm (Berkaloff et al. 1990; Ikeda et al. 2008), the *Navicula* 711 nm peak has a narrow bandwidth which is not representative of PSI emission, and in this study was principally attributed to FCP–PSII emission. The red shift of the Chl*a* Qy absorbance band in *Navicula* (Fig. 1c) could also signify an accumulation of Lhcf15-like antenna complexes. The Lhcf15 complexes isolated by Herbstová et al. (2017) contained fewer Chl*c* and Fx per Chl*a* as compared to the major FCP, which is reminiscent of the loss in accessory pigments observed in *Navicula* (Table 1; Fig. 1a). If the *Navicula* PSII antenna contains low energy Chl*a* species which emit at 711 nm, this may explain why no ~ 685 nm PSII emission was detected with FCP excitation, as the CP43 emitter may have been thermally inaccessible at 77 K. The relative difference in 77 K emission between the two species (Fig. 1b) was thus chiefly interpreted as *Navicula* having increased emission from the 695 nm PSII and 711 nm FCP emitters at the expense of emission from the 687 nm FCP–PSII emitter (and ostensibly its vibrational band at ~ 749 nm).

#### Changes in cell absorbance and emission in response to high light transitions

**Fig. 2:**
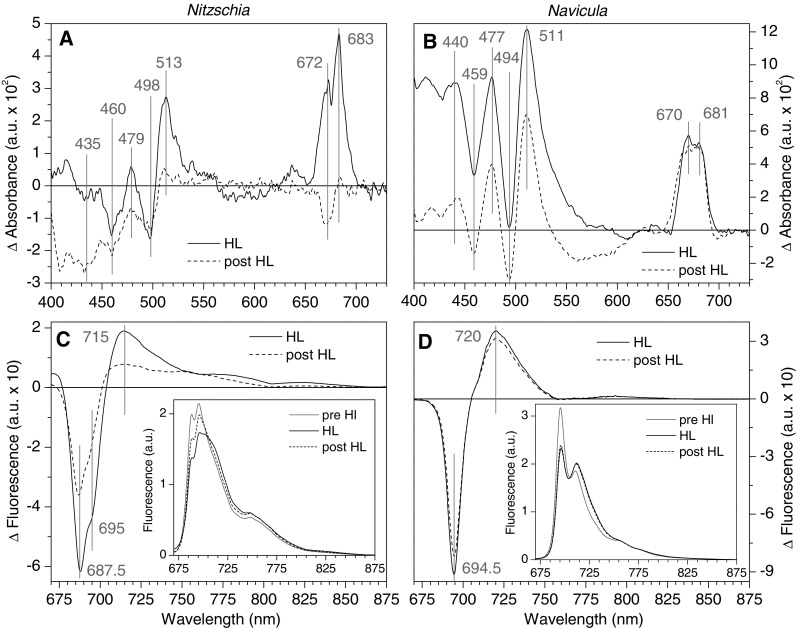
High light elicited changes in 77 K cell absorption and emission spectra. Spectra were collected from dark adapted cells prior to high light exposure (pre HL), cells exposed to 10 min 2000 µmol m^−2^ s^−1^ illumination (HL), and cells that were in the dark for 15 min following the HL exposure (post HL). HL minus pre HL (solid lines) and post HL minus pre HL (dashed lines) absorption difference spectra are shown for *Nitzschia* (**a**) and *Navicula* (**b**) cells. Absorption spectra were normalized to the Chl*a* 623 nm peak as in Fig. 1 prior to subtraction. Peak positions of interest are annotated with vertical grey lines. Difference spectra were calculated for *Nitzschia* from the average of three samplings from five separate cultures (*n* = 15) and in *Navicula* from the average of three samplings from four separate cultures (*n* = 12). HL minus pre HL (solid lines) and post HL minus pre HL (dashed lines) emission difference spectra are shown for *Nitzschia* (**c**) and *Navicula* (**d**) cells. Emission spectra (470 nm excitation) were normalized to integrated emission as in Fig. 1 prior to subtraction (see inserts). Peak positions of interest are annotated with vertical grey lines. Emission spectra are the mean from three separate cultures

The HL elicited changes in cell absorbance and emission are shown in Fig. 2; both species were treated to HL conditions (10 min at 2000 µmol m^−2^ s^−1^) that elicited the greatest steady state NPQ response (see "PSII performance during high light transitions"). In Fig. 2a,b absolute changes in absorbance magnitude at short wavelengths were interpreted with caution due to Rayleigh scattering effects in the frozen samples; yet since scattering loss in the red is much less, the magnitude of absorbance change within the red spectral region was read with high confidence. In both diatom species HL treated cells showed an increase in 77 K absorbance at ~ 512 nm compared to ~ 496 nm. An increase in room temperature absorbance at 512 nm in relation to 490 nm was reported in HL treated *P. tricornutum* cells, with the absorbance change attributed to an increase in DT:DD (Ruban et al. 2004). In both *Nitzschia* and *Navicula* there was an increase in low energy absorbance in the Chl*a* Qy region during HL, peaking at ~ 671 and ~ 682 nm (Fig. 2a, b). In comparison, Ruban et al. (2004) reported an increase in room temperature absorbance at 690 nm in *P. tricornutum* cells during HL. In higher plant thylakoids qE is associated with an increase in room temperature absorbance at ~ 685 nm (Ruban et al. 1992), which is also seen in vitro upon aggregation of LHCII trimers (Ruban and Horton 1992; Ruban et al. 1992; Kirchhoff et al. 2003). Likewise, when measured at 77 K aggregated-minus-trimeric LHCII absorption difference spectra were shown to be dominated by a strong positive peak at 680 nm (Ruban et al. 1997). This characteristic increase in low energy Chl*a* Qy absorbance has been advocated as a marker for the formation of Chl*a*–Chl*a* aggregates in LHCII during higher plant qE (Ruban et al. 1997, 2012).

77 K emission spectral shape was used to reveal the relative redistribution of FCP absorbed light energy between terminal emitters in response to HL (Fig. 2c, d). In *Nitzschia* cells there was a relative loss in emission from FCP and PSII at ~ 688 and ~ 695 nm; in *Navicula* fluorescence loss was centred at the ~ 695 nm PSII emitter. Both species showed a relative enhancement in emission > 710 nm. An analogous broad relative increase in far-red 77 K emission has been advocated as a marker for Chl*a*–Chl*a* quenching in aggregated LHCII complexes (Miloslavina et al. 2008; Andreeva et al. 2009), and for aggregation and pH induced quenching in isolated *Cyclotella meneghiniana* FCP complexes (Gundermann and Büchel 2012). The ~ 715/720 nm feature in Fig. 2c, d could possibly have contributions stemming from a relative enhancement in long wavelength FCP and/or PSI emission. The relative increase in ≥ 750 nm emission seen in *Nitzschia* was absent in *Navicula*, suggesting that this feature may be tied to the 687 nm emitter. Neither the HL absorption spectra nor the HL emission spectra contained any new derivative resolved bands (data not shown) as compared to those from the dark adapted cells in Fig. 1, implying that no new quencher species were formed during HL and that some level of quenching existed in dark adapted cells. These results illustrate that under NPQ conditions energy transfer is altered in a manner that preferentially hinders the transfer of excitation energy from antenna to PSII terminal emitter Chl*a*, but enhances exciton trapping on lower energy Chl*a* emittive states.

### PSII performance during high light transitions

#### Excitation pressure and NPQ

The cultured shoreline and slat flat species had the same capacity for photosynthesis as measured by ΦPSII in dark adapted cells (*Nitzschia* 0.578 ± 0.016 SD, *Navicula* 0.592 ± 0.025 SD) and φPSII in dark adapted cells that had been illuminated with growth irradiance for 10 min (*Nitzschia* 0.495 ± 0.024 SD, *Navicula* 0.495 ± 0.016 SD). Therefore, any inter-species differences measured in photoprotection should not be attributed to acclimation differences in respect to the culturing light environment, but can be primarily attributed to species-specific innate (genetic) adaptations.

**Fig. 3:**
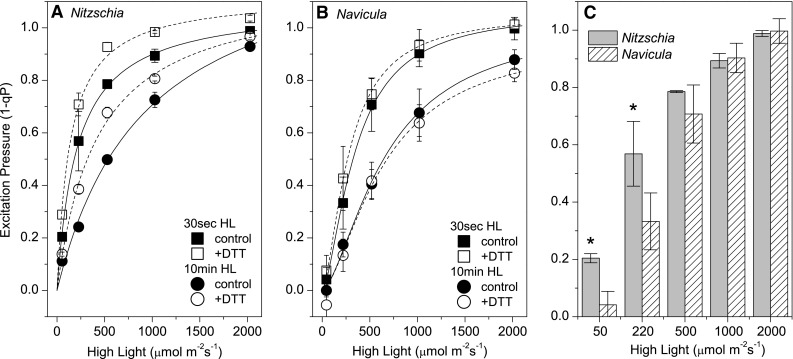
Excitation pressure generation in *Nitzschia* (**a**) and *Navicula* (**b**) cells. Excitation pressure at 30 s (square symbols) and 10 min (circle symbols) after the onset of high light are shown for control (solid symbols) and DTT pretreated (open symbols) (2.65 mM for *Navicula*, 5.3 mM for *Nitzschia*) cells. Solid and dashed lines represent Hill fits for control and DTT data sets, respectively (fit parameters given in Online Resource 2). **c** Excitation pressure at 30 s high light in control *Nitzschia* (grey columns) and *Navicula* (cross hatched columns) cells. Asterisks denote when 1 − qP in *Nitzschia* is significantly greater than *Navicula* (one-tailed two sample *t*-Test, *p* = 0.05). Error bars represent ± 1 SD of three separate cultures

The shoreline species *Nitzschia* had a larger effective absorbance cross section for excitation pressure generation (σ_1−qP_) than the salt flat species *Navicula*. During the initial (30 s) transition to low (50 µmol m^−2^ s^−1^) and moderate (220 µmol m^−2^ s^−1^) irradiances, significantly more excitation pressure was generated in *Nitzschia* than in *Navicula* (Fig. 3). The calculation of Km_1−qP_ values (HL irradiance level at half excitation pressure saturation), a larger Km_1−qP_ indicates a smaller *σ*_1−qP_, further suggested that *Nitzschia* had a larger basal *σ*_1−qP_ than *Navicula* (Online Resource 2). The enhanced conversion of photons to excitation pressure in *Nitzschia* could be a consequence of *Nitzschia*’s larger basal PSII antenna size (Table 1). During steady state HL conditions (10 min HL) excitation pressure relaxed in both species (Fig. 3a, b) and *σ*_1−qP_ dropped (by about one-quarter in *Nitzschia* and one-half in *Navicula*, as calculated by comparing Km_1−qP_ at 30 s and 10 min HL; Online Resource 2), suggesting that both species could downregulate excitation pressure generation during the shift towards photostasis.

**Fig. 4:**
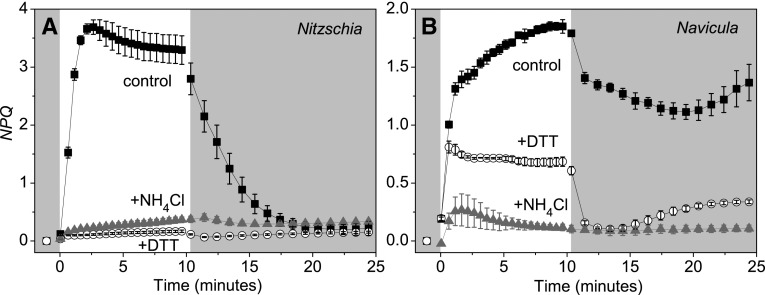
*NPQ* transients during a high light transition in *Nitzschia* (**a**) and *Navicula* (**b**) cells. The regions of the plot area with a darkened background correspond to dark conditions and the white portion of the background corresponds to 500 µmol m^−2^ s^−1^ illumination. Filled square symbol shapes, control cells. Open circle symbol shapes, cells pre-treated with DTT (2.65 mM for *Navicula*, 5.3 mM for *Nitzschia*). Grey triangle filled symbol shapes, cells pre-treated with NH_4_Cl (2.8 mM). Error bars represent ± 1 SD of three separate cultures

**Fig. 5:**
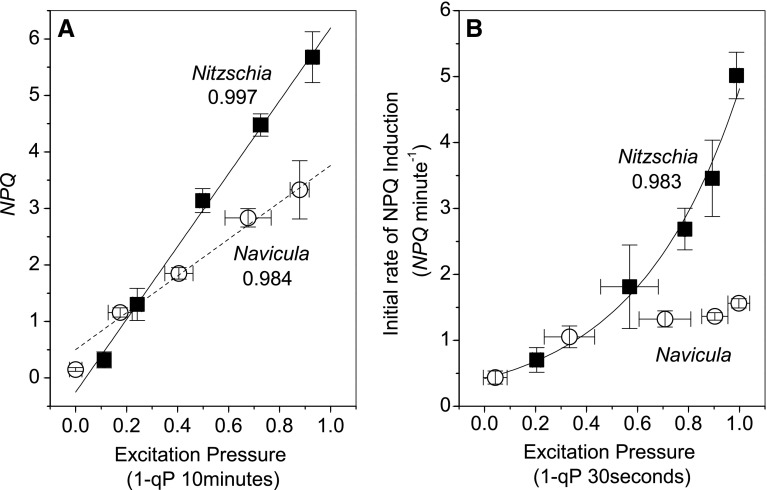
The dependence of NPQ on excitation pressure in *Nitzschia* (solid square symbols) and *Navicula* (open circle symbols) cells. **a** The magnitude of steady state *NPQ* measured at 10 min of high light illumination. **b** The initial rate of *NPQ* induction from 2 to 30 s upon the onset of high light illumination. Solid lines are fits of the *Nitzschia* data (linear in **a**, exponential in **b**) with corresponding *R*^2^ values; the dashed line is a linear fit of the *Navicula* data with corresponding *R*^2^ value. Error bars represent ± 1 SD of three separate cultures

The shoreline species generally exhibited a more dynamic and reversible photoprotective response than the salt flat species. Maximal *NPQ* amplitude in *Nitzschia* always developed within 3 min of HL illumination (Fig. 4a, Online Resource 3). To the contrary, in *Navicula* after the initial induction of NPQ with irradiances greater than growth light, *NPQ* continued to slowly rise throughout the HL illumination period (Fig. 4b, Online Resource 4). *Nitzschia* cells had a greater maximal capacity to dissipate excitation energy via NPQ, as witnessed by higher *NPQ* amplitude at the high excitation pressures generated by 500–2000 µmol m^−2^ s^−1^ HL (Fig. 5a, Online Resources 3, 4). *NPQ* induction rate had a positive exponential relationship with excitation pressure in *Nitzschia*, but plateaued at moderate HL intensities in *Navicula* (Fig. 5b). *Nitzschia* cells were more competent at regaining PSII function when transitioning from HL back to dark photolimiting conditions; quantum efficiency of PSII photochemistry and *NPQ* always exhibited a significantly greater post HL recovery in *Nitzschia* than what could be observed in *Navicula* (Table 2). Of note, *NPQ* recovery post HL never reached 50% in *Navicula* cells.

**Table 2:** Post high light recovery of PSII function

High light	φPSII recovery^a^		*NPQ* recovery^b^	
(µmol m^−2^ s^−1^)	*Nitzschia*	*Navicula*	*Nitzschia*	*Navicula*
50	0.979 ± 0.012*	0.915 ± 0.018	0.676 ± 0.077*	0.263 ± 0.036
220	1.006 ± 0.004*	0.832 ± 0.028	0.917 ± 0.056*	0.375 ± 0.030
500	0.984 ± 0.008*	0.733 ± 0.020	0.948 ± 0.040*	0.405 ± 0.041
1000	0.968 ± 0.018*	0.638 ± 0.044	0.952 ± 0.034*	0.382 ± 0.008
2000	0.928 ± 0.031*	0.562 ± 0.095	0.920 ± 0.064*	0.270 ± 0.074

A recovery value of 1.0 indicates full reversal of the effect from a 10 min high light exposure. Means ± 1 SD from three separate cultures

*Indicates *Nitzschia* values are significantly greater than *Navicula* (one-tailed two sample *t*-Test, *p* = 0.05)

^a^Calculated as: (maximal φPSII during 15 min post high light period)/ΦPSII

^b^Calculated as: (*NPQ* at end of 10 min of high light —minimal *NPQ* during 15 min post high light period)/(*NPQ* at end of 10 min of high light)

#### Influence of exogenous NPQ inhibitors

Pre-treatment of cells with DTT prevented ≥ 90% of *NPQ* amplitude from developing at all HL irradiances in *Nitzschia* (Table 3; Fig. 4a, Online Resource 3). Conversely, in *Navicula* non-photochemical quenching at growth light equivalence was not inhibited by DTT, and > 25% of *NPQ* amplitude was retained in the presence of DTT at all higher HL irradiances (Table 3; Fig. 4b, Online Resource 4). Dosage responses to DTT final concentrations of 0–21 mM (data not shown) revealed that DTT above 5 and 2.65 mM did not further inhibit NPQ induction in *Nitzschia* and *Navicula* cells, respectively, but did decrease ΦPSII. DTT pre-treatment increased excitation pressure generation in *Nitzschia* cells (Fig. 3a), and in turn, doubled steady state σ_1−qP_ (as calculated by comparing Km_1−qP_ at 10 min HL; Online Resource 2). DTT pre-treatment had no effect on excitation pressure generation in *Navicula* cells (Fig. 3b), implying that excitation pressure regulation in this species could be independent of DD de-epoxidation. The DTT resistant NPQ in *Navicula* had much faster HL on/off induction/relaxation kinetics than what was seen in the control cells (Fig. 4b, Online Resource 4).

**Table 3:** Inhibition of NPQ with DTT and NH_4_Cl

High light (µmol m^−2^ s^−1^)	*NPQ* at 10 min high light (treatment/control)			
	*Nitzschia*		*Navicula*	
	+DTT	+NH_4_Cl	+DTT	+NH_4_Cl
50	0.093 ± 0.064	0.369 ± 0.139	1.083 ± 0.192	− 0.177^a^ ± 0.100
220	0.063 ± 0.011	0.156 ± 0.028	0.364 ± 0.029	0.037 ± 0.005
500	0.053 ± 0.008	0.116 ± 0.008	0.372 ± 0.059	0.063 ± 0.012
1000	0.061 ± 0.004	0.132 ± 0.022	0.255 ± 0.010	0.072 ± 0.008
2000	0.088 ± 0.006	0.168 ± 0.011	0.259 ± 0.050	0.108 ± 0.032

Dark adapted cells were pretreated with DTT (2.65 mM for *Navicula*, 5.3 mM for *Nitzschia*) or NH_4_Cl (2.8 mM) prior to 10 min high light. Means ± 1 SD from three separate cultures

^a^Negative value indicates that NH_4_Cl treatment slightly enhanced fluorescence over the control

Ammonium chloride pre-treatment was used as a negative control for qE. Ammonium inhibited NPQ during HL illumination in both species (Table 3; Fig. 4, Online Resources 3, 4). The specificity of ammonium as an NPQ inhibitor over other chloride salts was verified against pre-treatments with equimolar NaCl and KCl solutions (data not shown). Ammonium inhibited ≳ 90% of *NPQ* amplitude in *Navicula* (Table 3), indicating that the majority of NPQ witnessed in *Navicula* was a type of qE. qE was divided into two subtypes: qE_XC_ for xanthophyll cycle dependent qE (i.e. DTT sensitive NPQ) and “qE_nonXC_” for xanthophyll cycle independent qE (i.e. DTT resistant NPQ). Photoinhibition was not believed to be a large contributor to the irreversible post HL NPQ observed in *Navicula* control cells, since the amplitude of *NPQ* in the qE inactivated + NH_4_Cl samples never approached that observed in the control samples throughout the duration of the HL transitions (Fig. 4b, Online Resource 4).

The qE_nonXC_ observed in *Navicula* could potentially have several origins, including: accumulated photodamage to PSII (photoinhibition), P700^+^ radical quenching within PSI (Schlodder et al. 2005), formation of triplet Chl*a* within the antenna (Ballottari et al. 2013), PSII reaction centre based quenching stemming from P680^+^ recombination reactions and/or cyclic electron transfer, or quenching facilitated by carotenoids (DT) which are already present or synthesized de novo. Antenna based qE causes fluorescence quenching when PSII reaction centres are closed (Fm quenching) and also when PSII reaction centres are open (Fo quenching) (Ruban et al. 2012). The DTT resistant NPQ in *Navicula* did not exhibit Fo quenching (Online Resource 5). Photoinhibition is another pathway that can quench Fm without quenching Fo, but was not anticipated to be a major contributor to the DTT resistant NPQ witnessed in *Navicula*. The DTT resistant NPQ was fully induced within ~ 30 s of HL illumination and relaxed 70–77% within 1 min of the transition from HL to dark conditions (Fig. 4b, Online Resource 4). These fast NPQ kinetics, especially in terms of quenching reversal, are highly inconsistent with photoinhibition, strongly suggesting that the DTT resistant NPQ witnessed in *Navicula* was predominantly not photoinhibition. Other examples of qE_nonXC_ have been reported in diatoms. A fast induced, DTT insensitive qE component conditional on pre-existing DT has been described in *C. meneghiniana* cells (Grouneva et al. 2008); PSII reaction center type of NPQ has been reported in *P. tricornutum* (Eisenstadt et al. 2008); pH dependent quenching without DD/DT conversion has been observed in vitro in PSII dimer-FCP complexes from several different diatom species (Yokono et al. 2015).

#### Apportionment of PSII excitation energy

**Fig. 6:**
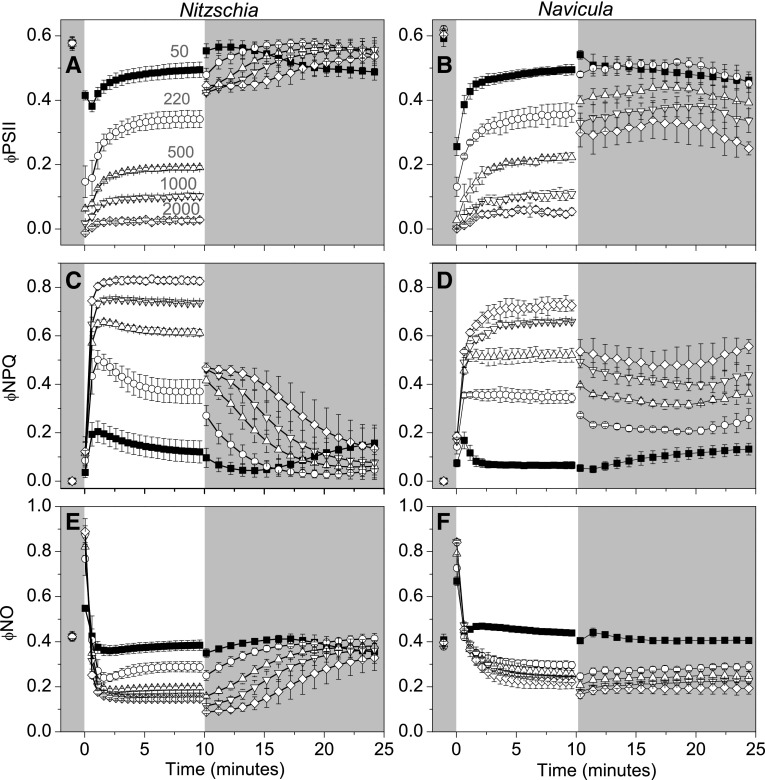
Quantum yields of PSII excitation energy conversion during high light transitions in *Nitzschia* (**a, c, e**) and *Navicula* (**b, d, f**) cells. The regions of the plot area with a darkened background correspond to dark conditions and the white portion of the background corresponds to high light illumination. In panel **a**, the magnitude of high light (in µmol m^−2^ s^−1^) applied during each trace in **a**–**f** is annotated in grey above its corresponding trace. Error bars represent ± 1 SD of three separate cultures

**Fig. 7:**
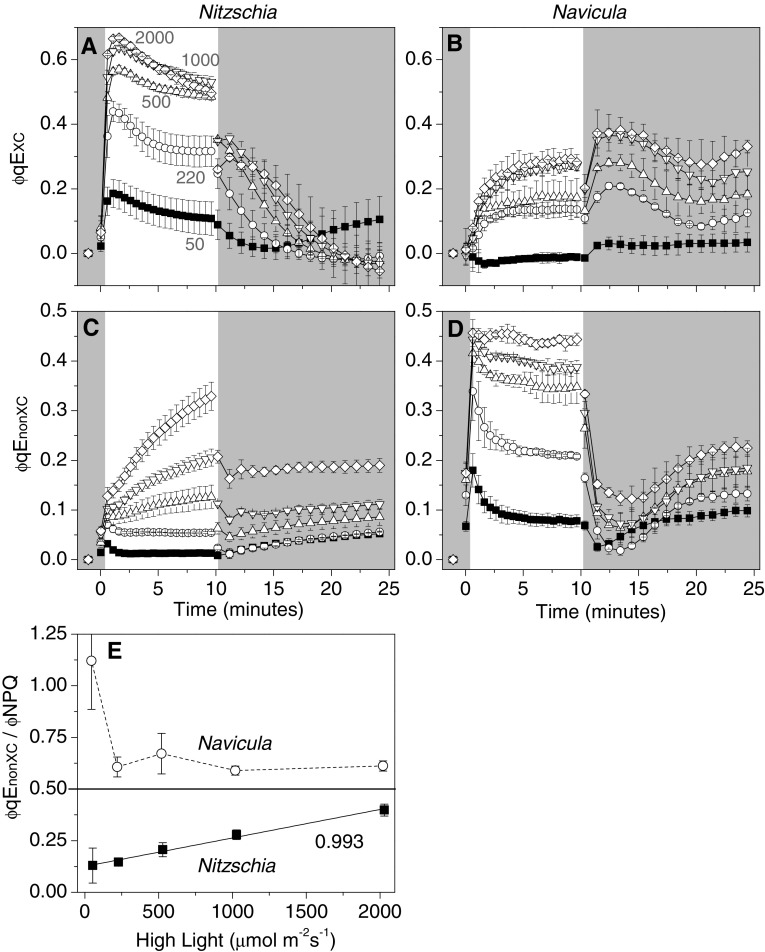
Quantum yields of NPQ energy dissipation during high light transitions in *Nitzschia* (**a, c**) and *Navicula* (**b, d**) cells. φNPQ was separated into DTT-sensitive (φqE_XC_) (**a, b**) and DTT-insensitive (φqE_nonXC_) (**c, d**) components. φqE_nonXC_ = φNPQ of cells pre-treated with DTT (2.65 mM for *Navicula*, 5.3 mM for *Nitzschia*). φqE_XC_ = φNPQ of control cells − φqE_nonXC_. The regions of the plot area with a darkened background correspond to dark conditions and the white portion of the background corresponds to high light illumination. In panel **a**, the magnitude of high light (in µmol m^−2^ s^−1^) applied during each trace in **a**–**d** is annotated in grey above its corresponding trace. **e** Relative contribution of energy dissipation via qE_nonXC_ to total energy dissipation via NPQ [ϕqE_nonXC_/(ϕqE_XC_ + ϕqE_nonXC_)] in *Nitzschia* (filled black square symbol shapes) and *Navicula* (open circle symbol shapes) cells. The solid line is a linear regression of the *Nitzschia* data with the corresponding *R*^2^ value. Error bars represent ± 1 SD of three separate cultures

The quantum efficiencies of photochemistry (φPSII), regulated non-photochemical dissipation (φNPQ), and constitutive thermal dissipation and fluorescence (φNO) sum the yields of PSII energy flux to unity (Hendrickson et al. 2004). φNPQ is presumably executed by qE, whilst φNO is used to describe non-regulated thermal dissipation and fluorescence from PSII cores. In both species φNO was important under conditions that do not elicit a strong qE response: when qE_XC_ would not yet be fully activated during the initial transition to all HL intensities, when low to moderate HL irradiances do not maintain high excitation pressures (nor presumably a high ∆pH), and under all HL intensities when qE_XC_ has been artificially inhibited by DTT pre-treatment (Fig. 6, Online Resource 6). φNPQ was experimentally split into two constituent components, φqE_XC_ and φqE_nonXC_ (Fig. 7). Pre-treatment of cells with saturating levels of DTT was used to inhibit qE_XC_, with any remaining regulated thermal dissipation credited to qE_nonXC_. The majority of regulated thermal energy dissipation in the shoreline species *Nitzschia* was via qE_XC_, whereas most of the regulated thermal energy dissipation in the salt flat species *Navicula* could be attributed to qE_nonXC_ (Fig. 7e). Likewise, rapidly activated NPQ energy dissipation was dominated by φqE_XC_ in *Nitzschia* and dominated by φqE_nonXC_ in *Navicula* (Fig. 7a–d).

#### Influence of electron flow on NPQ recovery

During measurement of HL transitions a slow increase in NPQ was typically witnessed in *Navicula* during the 15 min dark period that followed the 10 min illumination period (Fig. 4b, Online Resource 4). This “dark NPQ” could also be seen in *Nitzschia* following illumination with 50 µmol m^−2^ s^−1^ (Online Resource 3a). The induction of dark NPQ was slower than the induction of NPQ during HL. Dark NPQ was most evident in both species when cultures reached stationary phase, whereby dark NPQ was now also observable in *Nitzschia* following high irradiance illumination (Online Resource 7). The induction of dark NPQ was closely linked to a change in the redox equilibrium of the intersystem electron carriers: *NPQ* induction was consistently preceded by a decrease in φPSII and corresponded with changes in Kautsky fluorescence rise transients (Online Resource 7). Over-reduction in the redox state of the PQ pool under dark conditions was attributed to chlororespiratory electron flow. Chlororespiration would account for both (i) the reduced redox state of the PQ pool and (ii) acidification of the lumen and qE induction. NPQ attributed to chlororespiration has been previously reported in diatoms (e.g. Jakob et al. 2001; Cruz et al. 2011). The dark NPQ in *Navicula* seemed to retain a qE_nonXC_ component (Fig. 4b, Online Resource 4). Active dark chlororespiration would contribute to the lower φPSII observed in post HL *Navicula* cells (Table 2; Fig. 6b).

We suspect that the reversible qE_XC_ observed in *Nitzschia* was facilitated by a non-limiting supply of the DT epoxidase substrate NADPH, and conversely, the locked-in qE_XC_ observed in *Navicula* was due to a limitation in NADPH. Inhibition of photosynthetic linear electron flow during HL by pre-treatment with DCMU inhibited NPQ recovery in *Nitzschia* cells, much like what was seen in *Navicula* control cells (Online Resource 8). Interestingly, *Nitzschia* cells must have been capable of alternate ΔpH generating electron flow during HL, such as PSI cyclic electron transfer, for the activation of NPQ in the presence of DCMU, whilst in *Navicula* cells DCMU had an inhibitory effect on NPQ (Online Resource 8). Dark chlororespiration in *Navicula* would consume both of the co-substrates for DT epoxidase, NADPH and molecular oxygen, acting to inhibit qE_XC_ relaxation.

### Evaluation of photoprotective approaches

Both diatom species grew in a benthic state *in lab*. Cells readily settled to the bottom of the culture vessels after manual mixing. According to the growth form classification used by Barnett et al. (2015), *Nitzschia* would be best described as epipelon with *Navicula* as epipsammon. Bright field microscopy confirmed both species to be motile. A typical cell size (valve view) observed for *Nitzschia* was 15 × 40 µm and for *Navicula* was 6 × 10 µm; if including cell thickness (girdle view) and estimating cell shape as an elliptical cylinder, corresponded to cell volumes of 4700 and 380 µm^3^, respectively. When cultured with sediment *N. curvilineata* grows as a biofilm stabilized with extracellular polymeric substances (Sutherland et al. 2003); in our sediment-less culturing the cells also secreted mucilage (as seen with differential interference contrast microscopy) and formed a biofilm at the bottom of the culturing vessels. In stagnant *Nitzschia* cultures gas bubbles would sometimes become entrapped within the biofilm and cause some sections of the cell mat to break off and float on top of the water surface, as also observed by Sutherland et al. (2003); however these floating cells were not collected for experimental analysis. *Navicula* cultures did not noticeable accumulate mucilage and tended to adhere more to the surface of the culture vessels.

It is important to acknowledge that this study made no attempt to authentically replicate the natural light climates for *Nitzschia* and *Navicula*. Firstly, without field measurements the dynamics of their natural light environment could only be estimated based on habitat description. Secondly, the experimental HL transitions used abrupt changes in irradiance (from dark to full HL within a few milliseconds, and vice versa) that do not necessarily represent ecologically valid light transitions. Hence, the exact rates in the induction/relaxation and magnitudes of photoprotective energy dissipation described herein may not truly represent what would be observed in response to a natural HL transition. However, the relative comparison between irradiance intensities and between species can be considered appropriate for elucidating the underlying molecular mechanisms of NPQ and identifying the fast-acting photoprotective strategies for each species.

A benthic shoreline diatom, such as *Nitzschia*, living underneath churning water with a high particulate content would consistently be exposed to high amplitude (attenuated to full sunlight) and moderately fast periodicity (seconds–minutes) changes in irradiance (Raven and Geider 2003). Under the relatively low light laboratory culture conditions, this species produced a large basal FCP antenna for optimizing the collection of photons. However, this large antenna size lost its functional advantage when irradiance increased to levels which overwhelm photosynthetic electron transfer and sink capacity. The FCP light harvesting apparatus in *Nitzschia* overcame this conundrum by rapidly and reversibly converting itself to a photon energy dissipating parasol for PSII. qE_XC_ was seemingly obligatory for NPQ and the regulation of excitation pressure in this shoreline species, as treatment with DTT effectively inhibited the photoprotective response. The *Navicula* species was isolated from the soil surface of a salt flat where algae grow when shallow pools form after rain events (Kirkwood and Henley 2006). Diatoms in this barren landscape would be prone to less consistent high amplitude changes in light field as compared to their kin in a shoreline habitat. Despite being cultured under identical conditions as *Nitzschia, Navicula* cells had a smaller σPSII, fewer accessory pigments per Chl*a*, and a lower NPQ capacity—light harvesting traits befitting to a diatom adapted to a stable high irradiance light environ. Falkowski and Chen (2003) observed similar trends in σPSII based on the growth light intensity of diatom cultures and Lavaud et al. (2007) found higher NPQ capacities in diatoms from more turbulent habitats. The smaller antenna system in *Navicula* may be energetically favorable in a full sun environment; however, the reduced FCP antenna size limits the potential number of antenna based quenching sites when there is a strong photophysiological push to perform NPQ. Indeed, the difference in maximal *NPQ* amplitude observed between species corresponded to their difference in FCP accredited σPSII (*Navicula* max *NPQ* and σPSII was ~ 0.6 of *Nitzschia*). qE_nonXC_ which can activate in the absence of DD de-epoxidation is anticipated to be responsible for handling the routine irradiance fluctuations within *Navicula*’s native light environment that would stem from fast sun/cloud transitions, since all NPQ energy dissipation could be accredited to qE_nonXC_ when cells were rapidly transitioned from dark to growth light equivalent illumination (Fig. 7e, Online Resource 4a). Thus, qE in this slat flat diatom could be a largely passive phenomenon not requiring the metabolic investments (e.g. DD de-epoxidase, DT epoxidase, and the respective co-substrates ascorbate and NADPH) needed for large scale DD/DT cycling.

The present study can only speculate on the mechanism of the non-photochemical quenching observed in *Nitzschia* and *Navicula*. Further in-depth spectroscopic, biochemical, pigment, and genetic analyses would be required to distinguish molecular-level differences in NPQ and assign quenching loci. Likewise, since these two species are not described in the literature, much of this study’s conclusions are based on the results from other diatom species which would not have identical LHC protein composition and organisation. Of particular importance is how much, in addition to the antenna, the xanthophyll cycle components and the NPQ effector Lhcx proteins differ between these two species. For example in *Navicula*, a shortage of DD molecules, DD de-epoxidase and/or its co-substrate ascorbate could explain the lower NPQ amplitude, or conversely, a shortage of DT epoxidase or its co-substrates could be an underlying cause for the locked-in qE_XC_. As mentioned earlier, consumption of NADPH and oxygen by chlororespiration in *Navicula* could be a key cause for the sustained qE_XC_. Since NPQ has subtly different spectroscopic signatures between *Nitzschia* and *Navicula* (Fig. 2), the photophysical details of the quenching sites could also contrast. Consistent with quenching in *Nitzschia* being interconnected with DD/DT interconversion and Chl*a* aggregation, there was post HL relaxation of the 513 and 683 nm absorbance changes, respectively (Fig. 2a). Relaxation of qE_XC_ in *Navicula* seemed to be less tied to DT epoxidation, since a partial reversal of DT absorbance (as measured at 511 nm) did not induce equivalent-scaled reversals of the Chl*a* Qy absorbance change or excitation energy redistribution (Fig. 2b, d). This could suggest, similarly to the conclusions of Lepetit et al. (2010), the presence of distinct DT pools within the thylakoid. On the origins of qE_nonXC_ in *Navicula*, 77 K spectroscopic analysis of DTT treated cells would help decipher what contribution qE_nonXC_ has to the absorbance and fluorescence signatures observed in Fig. 2. Yokono et al. (2015) proposed that the functional uncoupling of PSII monomers under NPQ conditions can facilitate quenching even in the absence of DT. In the green algal *Chlamydomonas reinhardtii*, PSII–LHCII containing the Lhcx related Lhcsr3 can quench at low pH independent of xanthophyll cycle pigment conversion, with quenching postulated to stem from Chl-carotenoid interactions (Kim et al. 2017). Currently, we can at best assign the qE_nonXC_ observed in *Navicula* to a PSII reaction centre associated type of quenching. Of future interest is to identify the photophysiological purpose for the locked-in qE_XC_ in *Navicula*: does qE_XC_ primarily serve as a slow background photoprotective response for handling diel changes in light, is it involved in long-term photoprotective quenching during hypersaline stress conditions in the salt flat, or is it merely a by-product of chlororespiratory post HL metabolic repartitioning?

The role of non-qE photoprotective responses remains to be fully addressed in these two diatoms. The active contribution of cell motility, a behavioral response, was not investigated, although cell migration along the light field was probably minimal in this study due to the absence of a sediment substrate. If cell migration is an important aspect of the photoprotective response of *Navicula* within its natural habitat, this could mitigate the effect of having a limited amplitude fast acting NPQ (Blommaert et al. 2018). This study also did not specifically address the role of PSII protein turnover in photoprotection during longer duration HL stress (as in Wu et al. 2011, 2012; Lavaud et al. 2016); yet, within the short duration HL exposures used here, D1 turnover seemed to have no contribution. Pre-incubation of dark adapted cells with 0.4 g/L lincomycin had no effect on *NPQ* or PSII quantum yield throughout HL transitions (dark-to-10 min 500 µmol m^−2^ s^−1^ -to 15 min dark) (data not shown). Of note is that our photophysiological characterizations of *Nitzschia* and *Navicula* may not follow the same trends reported by other more encompassing studies on diatoms; such as motile epipsammic species exhibiting higher NPQ capacity than epipelic species among intertidal marine benthic diatoms (Barnett et al. 2015; Blommaert et al. 2018), and larger species experiencing lower susceptibility to HL stress than smaller species among marine centric diatoms (Key et al. 2010). Whether these discrepancies are a consequence of our study only investigating two species, or are an implication that originating light environment is the greatest determinant of photoprotective strategy remains to be answered.

## Electronic supplementary material

Below is the link to the electronic supplementary material.

Online Resource 1 77 K absorption spectra of isolated pigments normalized to absorbance maxima. Pigment abbreviations are as in main text. Chlc includes c1 and c2. *Nitzschia* cell extracts in 100% methanol were vacuum-evapor-concentrated on ice then spotted onto 250 μm polyester backed silica gel thin layer chromatography sheets (Whatman, GE Healthcare). Pigments were separated using 71.8 petrol ether: 18.6 ethyl acetate: 9.65 diethylamine (volume: volume) as the mobile phase. Individual pigment bands were excised from the thin layer chromatogram, dissolved in solvent, and separated from the silica by centrifugation. Samples in 1 dimethyl sulfoxide: 4 glycerol (volume: volume) were placed into the chamber of a copper cold finger and sandwiched between two quartz windows (the window proximal to the detector was frosted) with a sample optical path length of 5.0 mm. The cold finger was placed into a large radius Pyrex dewar (built *in house*) and submerged up to the quartz windows in liquid nitrogen. Compressed air was used to prevent condensation build up on the outside face of the dewar and the surrounding optics. The measuring beam was delivered to the sample in free space using the internal optics of the DW-2 spectrophotometer described in "Methods" —Supplementary material 1 (PDF 107 KB)

Online Resource 2 Fitting parameters for Fig. 3. Data sets were fit concatenate to the Michaelis–Menten function: *1–qP* = (*1–qP*_*maximal*_) (*irradiance*^*n*^) (*Km*^*n*^ + *irradiance*^*n*^)^–1^. *n* was initially determined from specie-specific global fits of each species’ data and then held fixed during the concatenate fitting—Supplementary material 2 (PDF 239 KB)

Online Resource 3 *NPQ* transients during high light transitions in *Nitzschia* cells. The regions of the plot area with a darkened background correspond to dark conditions and the white portion of the background corresponds to the time frame of 50 (panel A), 220 (panel B), 500 (panel C), 1000 (panel D), or 2000 (panel E) μmol m^–2^s^–1^ illumination. Filled square symbol shapes, control cells. Open circle symbol shapes, cells pre-treated with DTT (5.3 mM). Grey triangle filled symbol shapes, cells pre-treated with NH_4_Cl (2.8 mM). Error bars represent ± 1 SD of 3 separate cultures. Note y-axis scaling differs between panels—Supplementary material 3 (EPS 53 KB)

Online Resource 4 *NPQ* transients during high light transitions in *Navicula* cells. The regions of the plot area with a darkened background correspond to dark conditions and the white portion of the background corresponds to 50 (panel A), 220 (panel B), 500 (panel C), 1000 (panel D), or 2000 (panel E) μmol m^–2^ s^–1^ illumination. Filled square symbol shapes, control cells. Open circle symbol shapes, cells pre-treated with DTT (2.65 mM). Grey triangle filled symbol shapes, cells pre-treated with NH_4_Cl (2.8 mM). Error bars represent ±1 SD of 3 separate cultures. Note y-axis scaling differs between panels—Supplementary material 4 (EPS 164 KB)

Online Resource 5 Fluorescence quenching with open PSII reaction centres in *Nitzschia* (square symbol shapes) and *Navicula* (circle symbol shapes) cells without (closed symbol shapes) and with DTT pre-treatment (open symbol shapes) (2.65 mM for *Navicula*, 5.3 mM for *Nitzschia*). Quenching expressed as minimal fluorescence measured in the presence of far-red light at 10 minutes of high light illumination (*Fo′*) as percentage of minimal fluorescence measured in the presence of far-red light prior to high light illumination (*Fodark*). Error bars represent ±1 SD of three separate cultures—Supplementary material 5 (EPS 164 KB)

Online Resource 6 Quantum yields of PSII excitation energy conversion during high light transitions in DTT pretreated *Nitzschia* (A, C, E) (5.3 mM DTT) and *Navicula* (B, D, F) (2.65 mM DTT) cells. In panel A, the magnitude of high light (in μmol m^–2^ s^–1^) applied during each trace in A-F is annotated in grey above its corresponding trace. The regions of the plot area with a darkened background correspond to dark conditions and the white portion of the background corresponds to high light illumination. Error bars represent ± 1 SD of 3 separate cultures—Supplementary material 6 (PDF 348 KB)

Online Resource 7 Dark NPQ in *Nitzschia* (A, C) and *Navicula* (B, D) stationary phase cells. A and B, representative high light transitions illustrating the post illumination (indicated at the arrow) increase in *NPQ* (filled square symbol shapes) and the corresponding decrease in ϕPSII (open circle symbol shapes). The regions of the plot area with a dark background correspond to dark conditions and the white portion of the background corresponds to high light illumination. C and D, Kautsky fluorescence rise transients collected during the post high light drop in *NPQ* (at the data point directly preceding the arrow in A and B, trace in black), and during the post high light rise in *NPQ* (at the data point directly proceeding the arrow in A and B, trace in grey). Shown are the average traces from 9 separate cultures. Time zero on the x-axis corresponds to the start of the saturating light pulse. Baseline set to *Ft*, maximum normalized to *Fm’*. All panels, 500 μmol m^-2^s^-1^ high light—Supplementary material 7 (EPS 1614 KB)

Online Resource 8. The effect of DCMU on NPQ in *Nitzschia* (A) and *Navicula* (B) cells. Solid square symbol shapes, control. Solid circle symbol shapes, pretreated with 2.8 mM NH_4_Cl as a negative control for qE. Open upward triangle symbol shapes, pretreated with 4 μM DCMU. Regions of the plot area with a dark background correspond to dark conditions; regions with white background correspond to 500 μmol m^-2^s^-1^ illumination. Error bars represent ±1 SD, n = 3 separate cultures—Supplementary material 8 (EPS 90 KB)

## Abbreviations

Chl: Chlorophyll
DD: Diadinoxanthin
DT: Diatoxanthin
DTT: Dithiothreitol
FCP: Fucoxanthin chlorophyll protein
FWHM: Full width at half maximum
Fx: Fucoxanthin
HL: “High light”, i.e., illumination at an irradiance level that saturates the pre-existing photosynthetic capacity
LHC: Light harvesting complex
NPQ: Non-photochemical quenching
PS: Photosystem
qE: Non-photochemical excitation energy dissipation induced by energization of the thylakoid
qE_nonXC_: qE not directly dependent on xanthophyll cycle pigment conversion for induction
qE_XC_: qE directly dependent on xanthophyll cycle pigment conversion for induction
SD: Standard deviation of the mean
SE: Standard error of the mean
ΔpH: The trans-thylakoid proton gradient
σPSII: Effective PSII absorbance cross section
σ_1−qP_: Effective absorbance cross section for excitation pressure generation
~: Approximately
