# Supplementary figures and images for: Rapid regulation of excitation energy in two pennate diatoms from contrasting light climates
Source: Photosynth Res. 2018 Jul 14;138(2):149–65. doi: 10.1007/s11120-018-0558-0 (PMC6208626; doi:10.1007/s11120-018-0558-0)

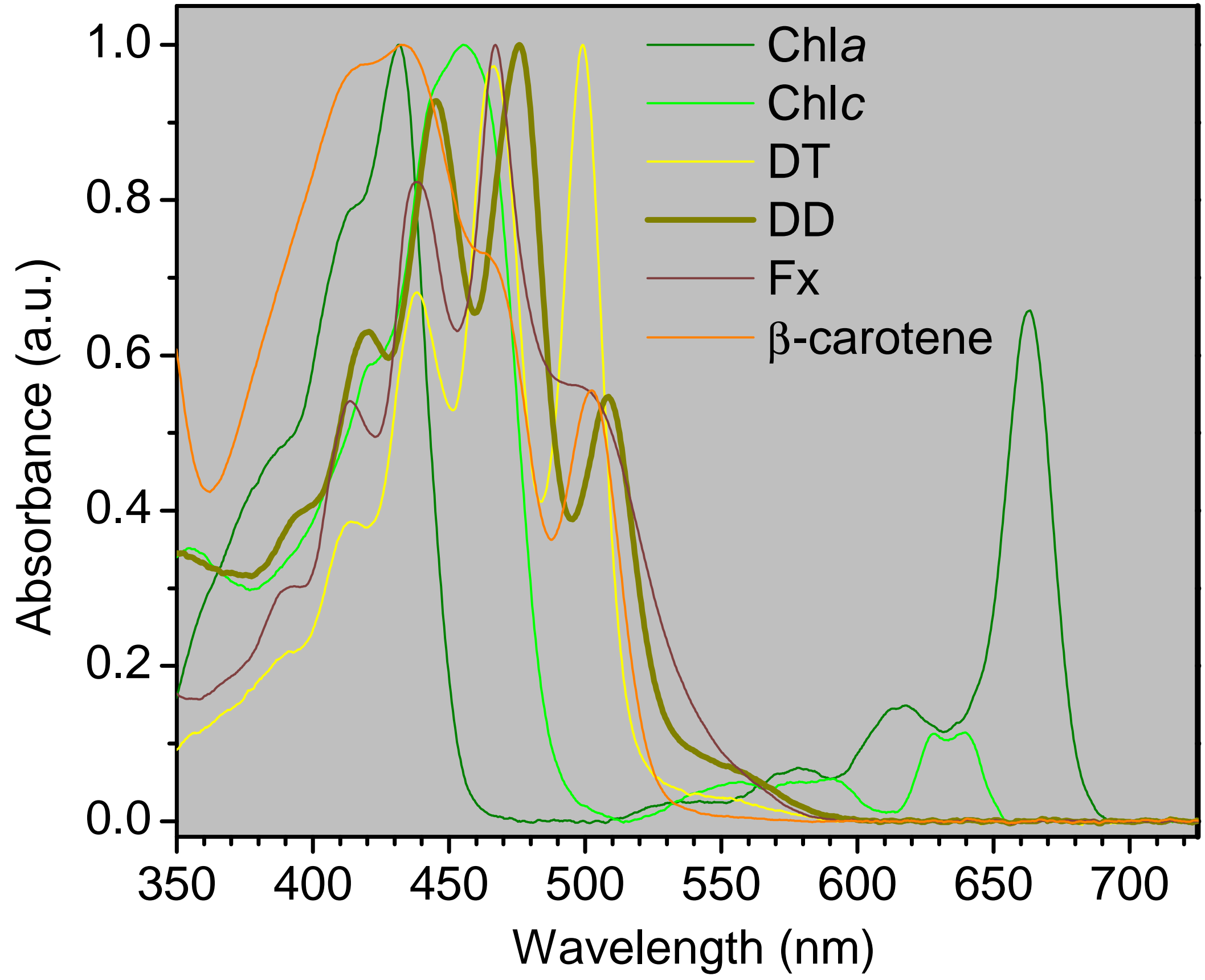

Supplement: Supplementary file 1 — Online Resource 1 77 K absorption spectra of isolated pigments normalized to absorbance maxima. Pigment abbreviations are as in main text. Chlc includes c1 and c2. Nitzschia cell extracts in 100% methanol were vacuum-evapor-concentrated on ice then spotted onto 250 μm polyester backed silica gel thin layer chromatography sheets (Whatman, GE Healthcare). Pigments were separated using 71.8 petrol ether: 18.6 ethyl acetate: 9.65 diethylamine (volume: volume) as the mobile phase. Individual pigment bands were excised from the thin layer chromatogram, dissolved in solvent, and separated from the silica by centrifugation. Samples in 1 dimethyl sulfoxide: 4 glycerol (volume: volume) were placed into the chamber of a copper cold finger and sandwiched between two quartz windows (the window proximal to the detector was frosted) with a sample optical path length of 5.0 mm. The cold finger was placed into a large radius Pyrex dewar (built in house) and submerged up to the quartz windows in liquid nitrogen. Compressed air was used to prevent condensation build up on the outside face of the dewar and the surrounding optics. The measuring beam was delivered to the sample in free space using the internal optics of the DW-2 spectrophotometer described in "Methods" —Supplementary material 1 (PDF 107 KB) [file 11120_2018_558_MOESM1_ESM.pdf]

*Nitzschia*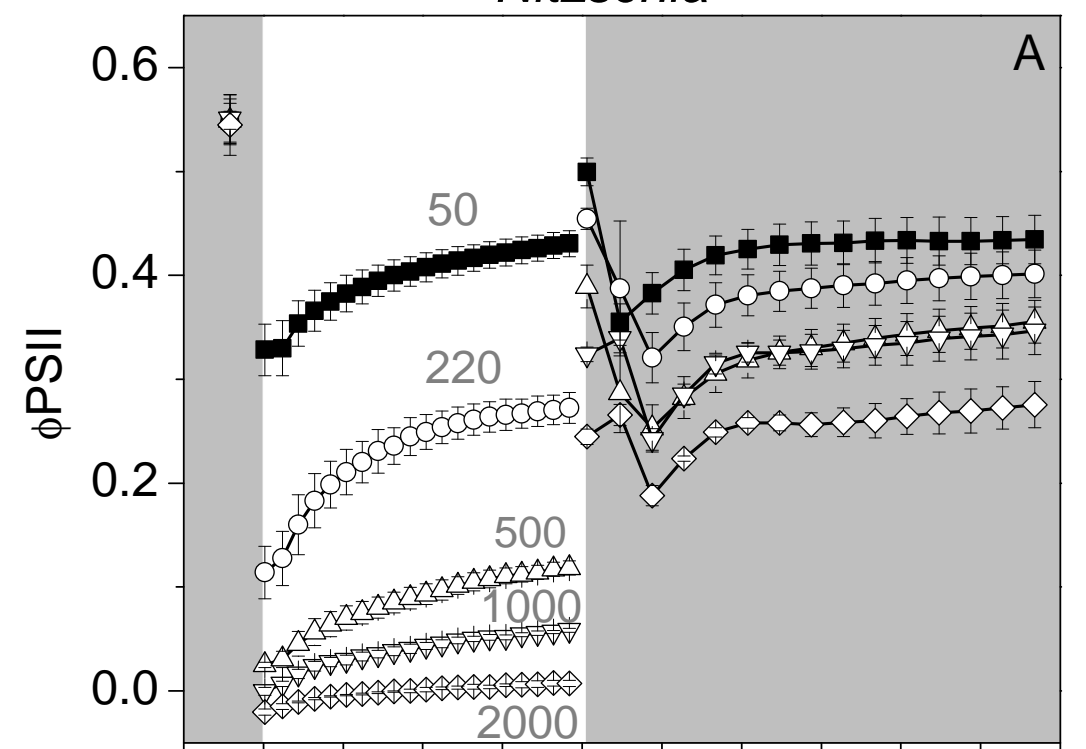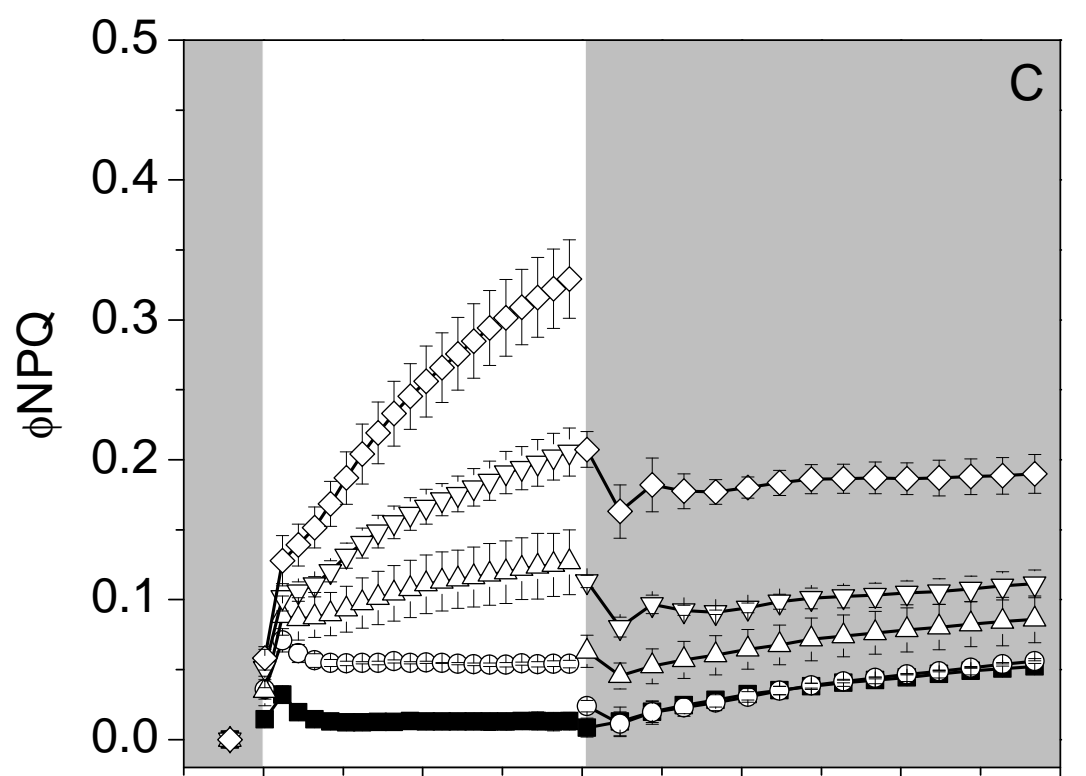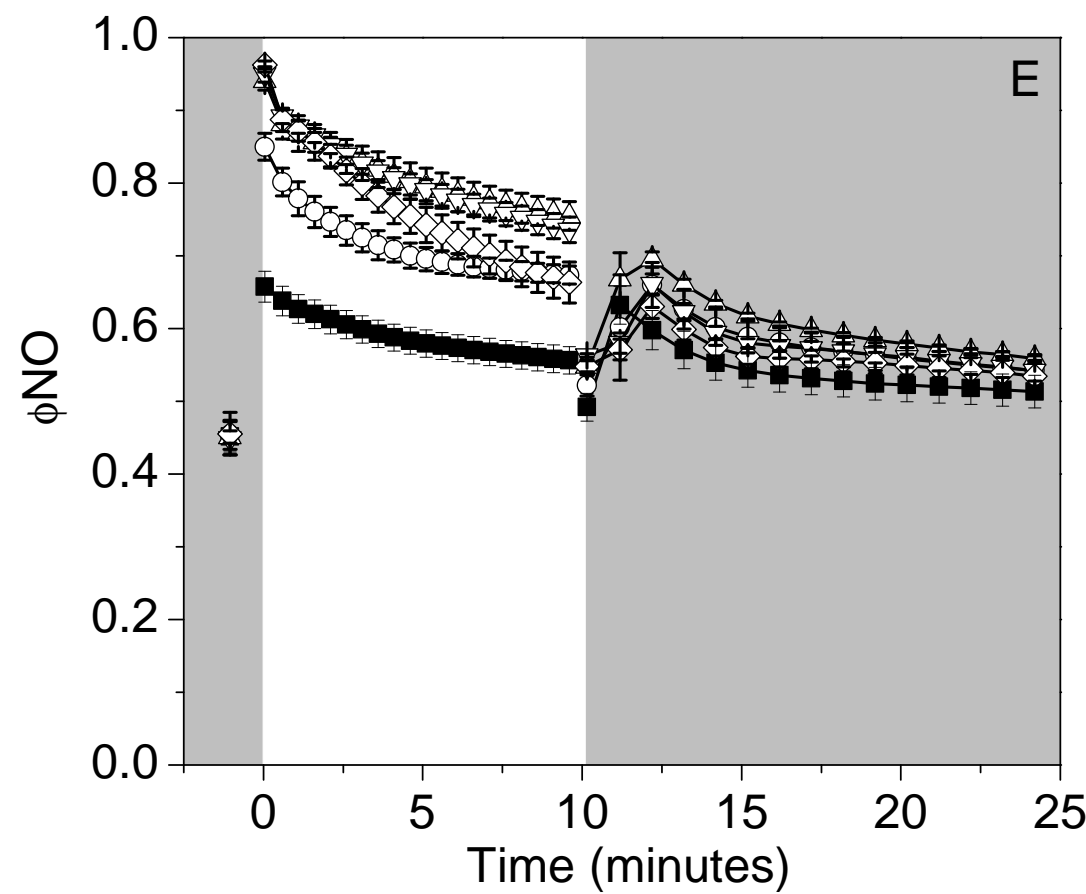*Navicula*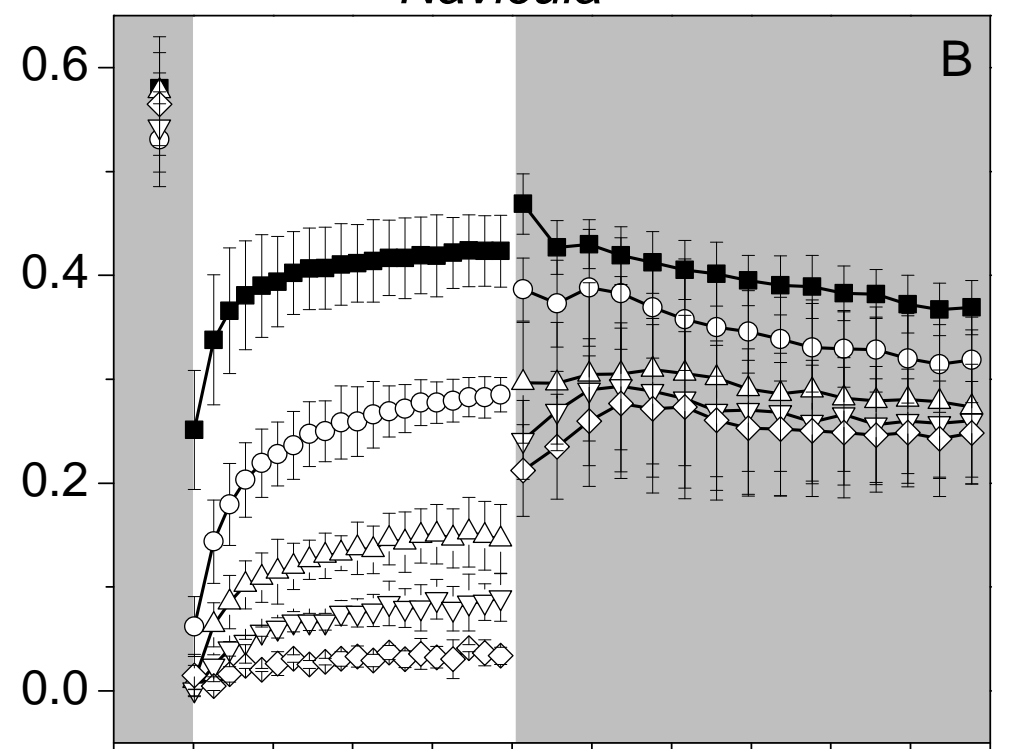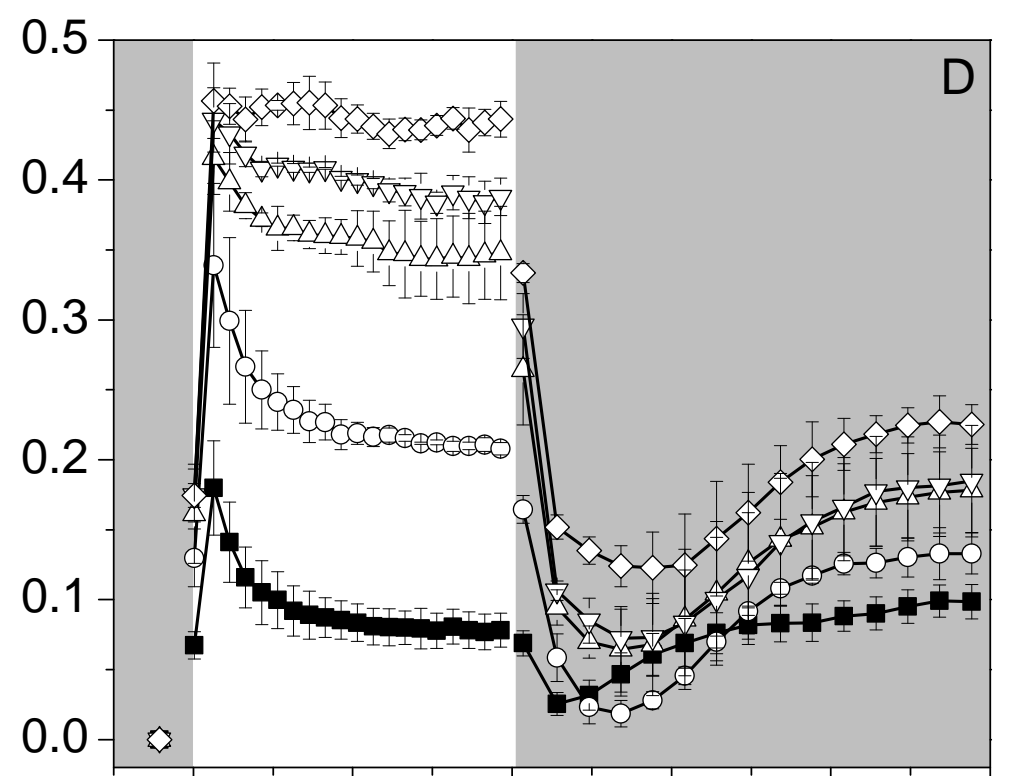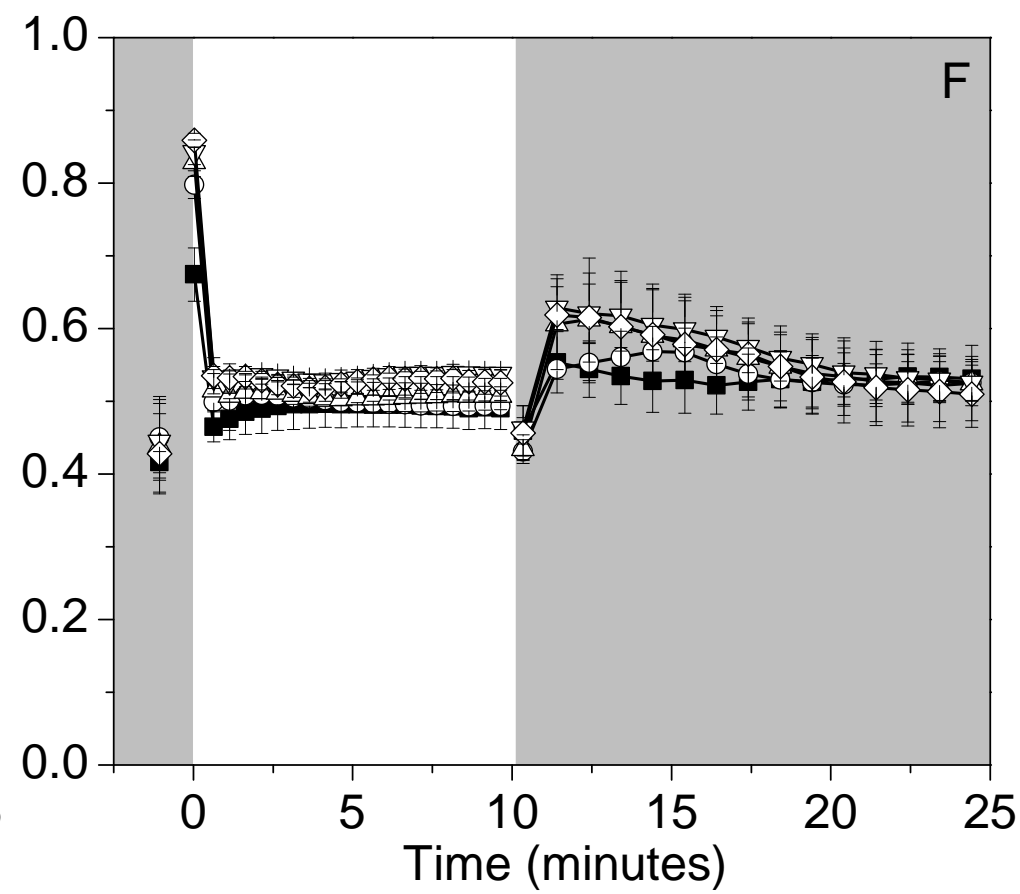

Supplement: Supplementary file 6 — Online Resource 6 Quantum yields of PSII excitation energy conversion during high light transitions in DTT pretreated Nitzschia (A, C, E) (5.3 mM DTT) and Navicula (B, D, F) (2.65 mM DTT) cells. In panel A, the magnitude of high light (in μmol m–2 s–1) applied during each trace in A-F is annotated in grey above its corresponding trace. The regions of the plot area with a darkened background correspond to dark conditions and the white portion of the background corresponds to high light illumination. Error bars represent ± 1 SD of 3 separate cultures—Supplementary material 6 (PDF 348 KB) [file 11120_2018_558_MOESM6_ESM.pdf]
