# Supplementary material for: Rapid regulation of excitation energy in two pennate diatoms from contrasting light climates
Source: Photosynth Res. 2018 Jul 14;138(2):149–65. doi: 10.1007/s11120-018-0558-0 (PMC6208626; doi:10.1007/s11120-018-0558-0)
Supplement: Supplementary file 2 — Online Resource 2 Fitting parameters for Fig. 3. Data sets were fit concatenate to the Michaelis–Menten function: 1–qP = (1–qPmaximal) (irradiancen) (Kmn + irradiancen)–1. n was initially determined from specie-specific global fits of each species’ data and then held fixed during the concatenate fitting—Supplementary material 2 (PDF 239 KB) [file 11120_2018_558_MOESM2_ESM.pdf]

Sample	Max 1-qP (±S.E.)	*Km* (µmol $m^{-2}s^{-1}$) (±S.E.)	*n*	Goodness of Fit (adj. R-square)
30 seconds HL				
Nitzschia control	1.09 (0.03)	215 (24)	1.00	0.974
Nitzschia +DTT	1.13 (0.02)	138 (9)	1.00	0.989
Navicula control	1.07 (0.04)	351 (34)	1.57	0.970
Navicula +DTT	1.056 (0.04)	285 (28)	1.57	0.969
10 minutes HL				
Nitzschia control	1.36 (0.05)	927 (80)	1.00	0.991
Nitzschia +DTT	1.17 (0.02)	425 (26)	1.00	0.994
Navicula control	1.03 (0.05)	669 (62)	1.57	0.977
Navicula +DTT	0.967 (0.061)	653 (82)	1.57	0.963
